# A systematic review of prenatally diagnosed vein of Galen malformations: prenatal predictive markers and management from fetal life to childhood

**DOI:** 10.3389/fped.2024.1401468

**Published:** 2024-07-03

**Authors:** Lavinia Di Meglio, Giordana Sica, Paolo Toscano, Giuliana Orlandi, Luigi Manzo, Laura Letizia Mazzarelli, Carmine Sica, Aniello Di Meglio

**Affiliations:** ^1^ Residency School of Pediatrics, University of Rome Tor Vergata, Rome, Italy; ^2^ University of Medicine and Surgery Luigi Vanvitelli, Naples, Italy; ^3^ Department of Neuroscience, Reproductive Sciences and Dentistry, School of Medicine and Surgery Federico II of Naples, Naples, Italy; ^4^ Diagnositica Ecografia e Prenatale di A. Di Meglio, Naples, Italy

**Keywords:** vein of Galen malformations, vein of Galen aneurysmal malformations, fetus, prenatal diagnosis, VGM, VGMs, VGAM, VGOM

## Abstract

**Introduction:**

Vein of Galen malformations (VGMs) account for less than 1% of all intracranial vascular malformations. However, in fetal and pediatric populations, they represent the most common vascular malformation of the brain. For the effective management of this condition, an optimal knowledge of its prenatal and postnatal clinical features is mandatory.

**Methods:**

Articles published between 1 January 2003 and 31 January 2024, reported in PubMed and EMBASE, were evaluated for a systematic review analyzing the prenatal and postnatal features and management of fetal VGMs.

**Results:**

Thirty-one papers reporting information on 51 prenatally diagnosed VGMs were included. The most common prenatal features were fetal hydrocephalus (39%) and cardiomegaly (56%). Postnatal data for 43 VGM cases are described. The overall mortality was 58.14%. In total, 77.78% of the survivors had normal development.

**Conclusions:**

Close follow-up and a multidisciplinary approach are mandatory to manage this condition. Our study aimed to provide a guide for gynecologists, neonatologists, cardiologists, and neuroradiologists.

## Introduction

Vein of Galen malformations (VGMs), also known as vein of Galen aneurysmal malformations (VGAMs), are extremely rare congenital arteriovenous malformations involving the intracranial vessels. The incidence of VGMs is 1:10,000–25,000, accounting for less than 1% of all intracranial vascular malformations (1, 2). However, in fetal and pediatric populations, VGMs represent the most common intracranial vascular anomaly, accounting for 30% of all endocranial vascular malformations (1–3).

VGMs involve multiple arteriovenous shunts draining into a dilated vein called the prosencephalic vein of Markowski (MProsV), which normally disappears during embryogenesis and represents the precursor of Galen's vein. During neurogenesis, in the choroidal stage between the 6th and 11th weeks, the choroid plexus supplies the developing brain, and the MProsV represents the main draining vessel. Later, with the development of the main cortical arterials and cerebral veins, the choroidal arteries and the anterior part of the MProsV disappear, with its posterior segment forming Galen's vein. The genesis of VGAM arises from an anomaly of this process, where the anterior part of the MProsV does not disappear but enlarges due to the high pressure of the choroidal feeders, forming a vascular malformation (2–6). The diagnosis can be achieved prenatally, soon after birth (also defined as “neonatal VGM,” with early development of heart failure) or later (also defined as “infant type,” with the development of neurological signs and symptoms such as seizure, macrocephaly, and hydrocephalus).

According to the Lasjaunias classification, VGMs can be divided into choroidal type and mural type (5, 7).

The choroidal type is the most frequent and severe: multiple feeding arteries form a nidus before entering the anterior part of the MProsV. The feeders are all choroidal arteries, and due to the multiple high-flow fistulas, this type is associated with a neonatal presentation and is more prone to causing heart failure. In addition, this type represents most of the prenatally diagnosed VGMs. The mural type is characterized by single or multiple fistulas that drain directly into the wall of the MProsV. Compared to the choroidal type, the smaller number of fistulas are less prone to induce precocious cardiac failure; however, the mural type is more prone to producing a larger dilatation of the MProsV, leading to macrocephaly and hydrocephalus in infancy (7).

In this paper, we conducted a retrospective study and performed a systematic literature review. Our study aimed to investigate the role of prenatal diagnosis of VGMs, identify prenatal predictive features, and define the best prenatal and postnatal approach for the management of VGMs.

## Methods

**Figure 1:**
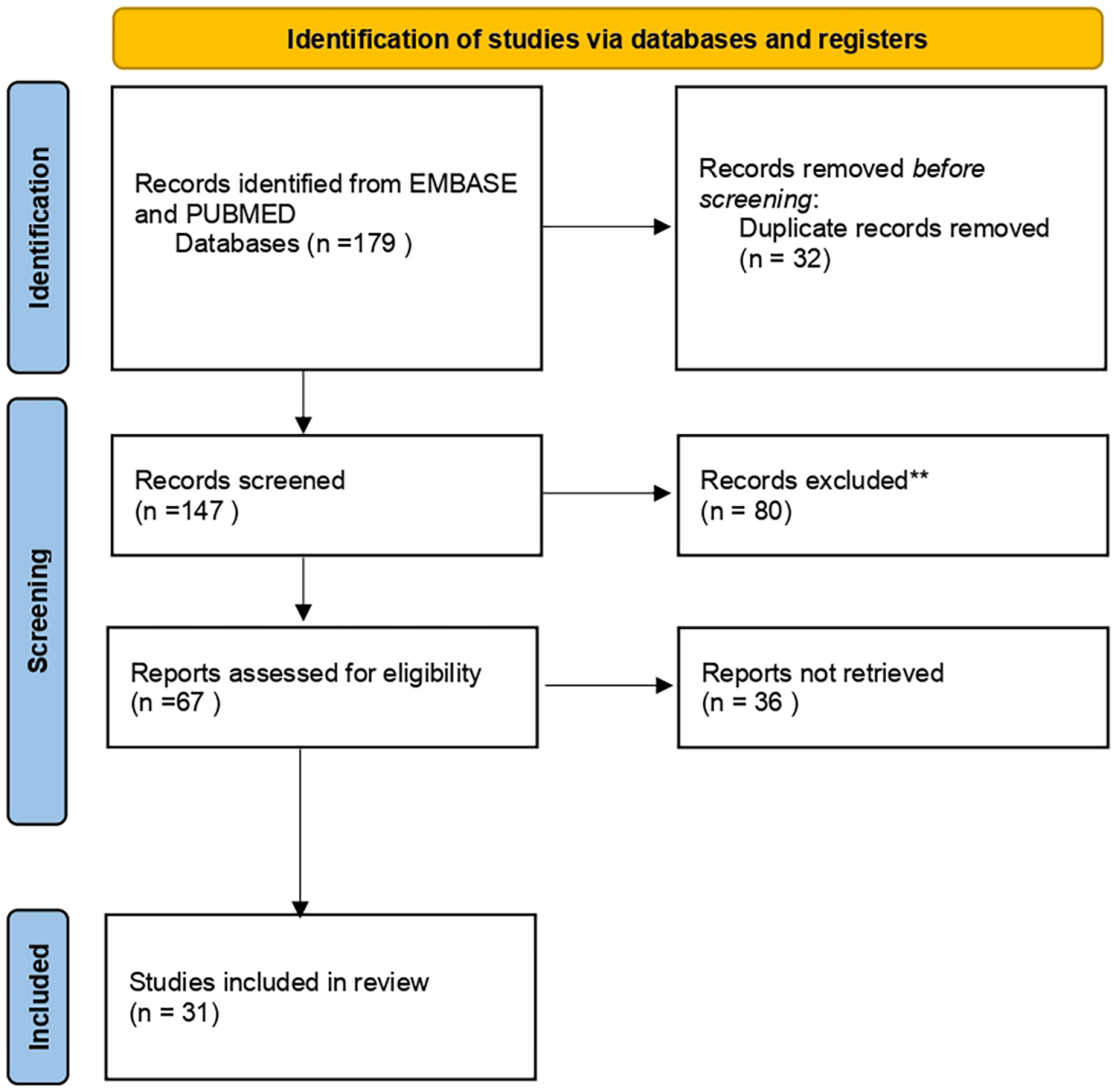
PRISMA chart.

We conducted a systematic review in PubMed and EMBASE from 1 January 2003 to 31 January 2024 using the following keywords: fetal vein of Galen aneurysmal malformation, fetal VGAM, arteriovenous malformation of Galen vein, fetal Vein of Galen malformation, and prenatal diagnosis. We searched for papers describing prenatally diagnosed VGMs. Only case reports and case series were included. In total, 179 articles were selected. Data extraction from individual studies was performed in triplicate (LDM, LLM, and GS). After reading titles and abstracts and eliminating duplicates, 31 papers were selected and assessed by the authors (Figure 1). We assessed the gestational age (GA) at diagnosis, the presence of prenatal hydrocephalus, cardiomegaly, associated anomalies, the pregnancy outcomes, and birth and postnatal outcomes. In each included paper, we evaluated all variables. For each variable, we reported the total number of cases in which that variable was available; when a variable was not described, it was considered “not valuable.” All data were collected in a dedicated database and analyzed by a statistician using SPSS for Windows (version 23.0, SPSS Inc., Chicago, IL). Categorical data are presented as numbers and percentages, and continuous data are reported as mean/median and range, according to their statistical distribution.

## Results

Thirty-one papers reporting 51 cases were included (8–38). All pregnancies were singleton except for one twin pregnancy. Data regarding prenatal and birth outcomes are depicted in Table 1. The diagnosis was performed around the 31st week of GA (22–37). Fetal hydrocephalus was present in 39.21% (20/51), cardiomegaly in 56.86% (29/51), and fetal heart failure was present in 65.51% (19/29) of fetuses with cardiomegaly. Fetal MRI was performed in 62.79% (76/43) of cases. The associated anomalies included two cases of polyhydramnios, one case of oligohydramnios, one case of vertebral defects, anal atresia, cardiac defects, tracheo-esophageal fistula, renal anomalies, and limb abnormalities (VACTERL) syndrome, one case of recurrent hydrothorax, and one case of adrenal hemorrhage. We have postnatal data for 43 VGAM cases (4 cases opted for voluntary termination of pregnancy, and no data regarding postnatal care were present in 4 cases). The median GA at birth was 38 weeks (28–41). The postnatal outcomes are depicted in Table 2. In total, 23.26% (10/43) were discharged home in stable condition, with the indication of elective embolization at 5 months. Of these, four received elective embolization: in three cases, the embolization was successful and uneventful; in one case, embolization was complicated by secondary hemorrhage, resulting in the patient developing hemiparesis. Of the 10, 1 experienced spontaneous thrombosis, 2 developed hydrocephalus and seizure at 3 months, and 1 developed hydrocephalus at 2 months, requiring urgent embolization, which resulted in a full recovery. Two of the 10 were still waiting for elective embolization. We found that 78.53% (33/42) were admitted to the neonatal intensive care unit (NICU), with the median day of admission being the first day of life (1–15). Heart failure was the main cause of NICU admission (Table 2). All these patients received cardiovascular therapy. Of these, 20 died, with their condition being too severe to perform embolization; the median day of death was 4th day. In 12 cases, urgent embolization was performed, and 7 patients survived after the procedure. In one case, embolization was not performed, and the patient survived with severe neurocognitive development delay. A ventriculoperitoneal shunt was positioned in four patients. The overall mortality was 58.14% (25/43). In the survivors’ group, 77.78% (14/18) had normal development, 11.10% (2/18) had a mild neurological disability (mild psychomotor impairment and right hemiparesis that improved after physiotherapy and occupational therapy), and 11.10% (2/18) presented with severe neurological disability. Death occurred due to heart failure and brain injury. Table 3 depicts the difference between the fetal features of VGM patients with favorable outcomes (normal or mild cognitive impairment) and those with unfavorable outcomes (exitus or severe cognitive impairment). The presence of prenatal cardiomegaly, heart failure, and hydrocephalus is associated with a poor outcome.

**Table 1:** Prenatal and birth features.

Variable (*N*)	Variable subclassification	Results
GA at presentation (*N* = 50)		31 weeks (22–37)
VTP (*N* = 51)		7.84% (4/51)
Prenatal hydrocephalus (*N* = 51)		39.21% (20/51)
Cardiomegaly (*N* = 51)		56.86% (29/51)
Fetal heart failure (*N* = 51)		37.25% (19/51)
MRI *in utero* (*N* = 43)		62.79% (27/43)
GA at birth (*N* = 43)		38 week (28–41)
	Extremely preterm	4.67% (2/43)
	Very preterm	2.33% (1/43)
	Preterm	23.25% (10/43)
	At term	69.75% (30/43)
Mode of delivery (*N* = 35)	Cesarean section	68.57% (24/35)
	Vaginal delivery	28.57% 10/35)
	Operative delivery	2.86% (1/35)
Apgar score (*N* = 29)	Apgar 10' 7–10	75.86% (22/29)
	Apgar 10' 4–6	24.14% (7/29)
	Apgar 10' 0–4	0% (0/29)

VTP, voluntary termination of pregnancy.

**Table 2:** Postnatal features.

Variable (*N*)	Variable subclassification		Results	
Newborns with VGM (*N* = 43)	Discharged home with elective embolization		23.26% (10/43)	
	Admission to NICU		76.74% (33/43)	
Main complication for NICU admission (*N* = 33)	Heart failure		88.88% (29/33)	
	Hydrocephalus		3.03% (1/33)	
	Respiratory distress		6.06% (2/33)	
	Sepsis		3.03% (1/34)	
Day of admission to NICU (*N* = 28)			1st day of life (1–15)	
Outcome in NICU (*N* = 33)	Embolization (*N* = 12)	Exitus	36.36% (12/33)	41.66% (5/12)
		Survivors		58.34% (7/12)
	Medical therapy (*N* = 21)	Exitus	63.64% (21/33)	95.23% (20/21)
		Survivors		4.77% (1/21)
Outcome of newborns discharged home with elective embolization (*N* = 10)	Waiting for elective embolization		20% (2/10)	
	Spontaneous thrombosis		10% (1/10)	
	Emergent embolization		30% (3/10)	
	Elective embolization		40% (4/10)	
Outcome of VGM newborns (*N* = 43)	Exitus		58.14% (25/43)	
	Survivors		41.86% (18/43)	
Day of death (*n* = 18)			4th day of life (1–26)	
Outcome of survivors (*N* = 18)	Normal neurodevelopment		77.78% (14/18)	
	Mild NDD		11.10% (2/18)	
	Severe NDD		11.10% (2/18)	

NDD, neurodevelopmental delay.

**Table 3:** Prenatal features in good and poor postnatal outcomes.

Variable	Survivors with none or mild NDD *N* = 16	Exitus or severe NDD *N* = 27	*p*-value
Mean GA at diagnosis (weeks)	33	32	0.012
Prenatal cardiomegaly	4/16	18/27	0.012*
Prenatal heart failure	2/16	14/27	0.02*
Prenatal hydrocephalus	0/16	13/27	0.0006**

NDD, neurodevelopmental delay.

* stand for *p* value <0.05 and ** *p* value <0.01.

## Discussion

VGMs account for less than 1% of all intracranial vascular malformations; however, in fetal and pediatric populations, they represent the most common vascular malformation of the brain [(1, 2)]. The diagnostic process starts in fetal life, and a multidisciplinary approach is needed to diagnose and treat this condition.

The features of the VGM and its complications, mainly cardiopulmonary failure and encephalomalacia, are not the same in the fetus and in the newborn. Early diagnosis before birth has multiple benefits: the mother can receive adequate information, a specific postnatal team can be consulted prenatally, and the parents can be fully informed and able to organize postnatal care.

### Prenatal management

Prenatal diagnosis is achieved in almost 30% of cases (1). It is usually made during the third trimester. In the sagittal plane, the ultrasound appearance of a VGM is characterized by a hypoechogenic midline tubular structure localized in the posterior part of the third ventricular, described as a “comet tail” or “keyhole sign.” Doppler imaging shows turbulent arterial and venous flow (1, 18). In the coronal plane, a VGM appears as a round cystic structure. The differential diagnosis includes arachnoid cysts, porencephalic cysts, choroidal papillomas, and brain tumors; the use of color Doppler can easily help differentiate these conditions (1, 18, 19, 21, 23, 26, 29). The two most common prenatal features are fetal hydrocephalus and fetal cardiomegaly.

Hydrocephalus is present in almost 40% of cases and results either from high venous pressure, which interferes with the cerebrospinal fluid (CSF) drainage, or from the compressive effect of the malformation. In our study, the presence of prenatal hydrocephalus was associated with a poor outcome (15, 39).

Cardiomegaly is present in almost 60% of cases. The increment of the cardio thorax index is mainly due to the dilatation of the superior vena cava and the right ventricle, probably due to the high venous return from the brain (40). Cardiac involvement is less pronounced in fetal life compared to the postnatal period due to the low vascular resistance of VGMs being balanced by the low resistance of the uteroplacental unit (3, 41).

A prenatal neuroradiological, cardiological, and neonatological evaluation should be offered to explain the possible complications and postnatal management to the pregnant woman. We also advise a genetic consultation. A fetal ultrasound with fetal echocardiography should be offered at least every 2 weeks (3, 39, 40). The type of birth should be personalized for each case; in the absence of cardiological dysfunction and no other contraindications, vaginal delivery can be performed safely (1). From our review, vaginal delivery was performed in almost one-third of cases.

In previous studies, the presence of fetal cardiomegaly and ventriculomegaly were associated with poor outcomes (3, 40, 42, 43). Our prenatal data overlaps with those from previous studies; in our review, the presence of prenatal cardiomegaly, heart failure, and hydrocephalus was associated with a worse postnatal outcome, and none of the newborns discharged home with elective embolization had presented these conditions prenatally. When these features are present, we recommend delivering the newborn in a fully equipped center.

When there is a suspicion of a VGM, performing a fetal MRI should always be advised for a better study of brain anatomy and to predict the clinical outcome (17, 32, 33). The role of MRI has many clinical impacts. First, MRI can better define the VGM anatomy and its dimensions; in addition, it gives information about the presence of hydrocephalus and the development of brain parenchyma, including the presence of diffuse brain injury or hemorrhage, which may impact future clinical management. Arko et al. have shown that in fetal and postnatal MRI, the mediolateral diameter or cross-sectional area of the straight or falcine sinus at its shortest section serves as a predictor of neonatal mortality and an indication for intervention. The lesser constriction of this point was associated with neonatal-onset VGMs and thus a more severe outcome (44). Saliou et al. described how the presence of “pseudofeeders” and increased CSF volume, defined by an Evans index of >0.31, are predictive of the development of heart failure after birth and of a worse outcome. Pseudofeeders are the cortical branches of the MCA and are not choroidal arteries; they supply the brain parenchyma and are not the vessels involved in the VGM (45). The prenatal MRI features have not been included in this review due to a lack of data.

From the latest discoveries, the prenatal MRI findings, such as diffuse parenchymal brain injury or hemorrhage, dilatation of the falcine sinus at its shortest section, and the presence of pseudofeeders or an Evans index of >0.31, play a pivotal role in the decision algorithm (44–46).

### Postnatal management

At birth and during the first hours or days of life, patients with VGMs usually present in good and stable condition. However, instability can develop within hours or days, mainly due to the transition from fetal to postnatal circulation and the type of VGMs. The choroidal type tends to be diagnosed in the newborn due to its hemodynamic impact, while the mural type may be diagnosed after the neonatal period. Newborns with VGMs should be assisted or rapidly transferred to a specialized center where a neuroradiologist, neonatologist, pediatric cardiologist, and neurosurgeon are present (43).

Embolization is the main treatment for VGMs: the approach can be transarterial, transvenous, or both, but transarterial close is the most commonly used method. The transvenous approach is less effective and carries a higher risk of complications (31, 44, 47). Usually, more than one procedure is needed to achieve the occlusion of the malformation, as the amount of parenteral fluid and contrast is limited in newborns and infants (11). Various agents and devices can be used for embolization, including N-butyl cyanoacrylate (NBCA) glue, liquid embolic agents, and coils, with liquid glue being the most frequently used. The main goals of embolization are to reduce cardiovascular stress and high pressure on the brain to favor CSF drainage and allow normal brain development. The timing of embolization is governed by cardiac hemodynamics and the extent of brain involvement (11, 43, 47). Other techniques are microsurgery, radiosurgery, or a combination of several procedures (11, 31, 44, 47).

Lajaunias has proposed “The Bicetre score,” which assesses the cardiological, neurological, hepatic, renal, and respiratory function. If the score is <8, the condition is too severe for embolization; a score between 8 and 12 indicates that the patient is a candidate for urgent treatment, while a score >12 suggests elective treatment should be proposed if the patient is at least 5 months old (43, 48). This score is, however, not yet used, and there is always more focus on prenatal and postnatal predictive markers, including the presence of dilatation of the falcine sinus at its shortest section, the presence of pseudofeeders or an Evans index of >0.31, and prenatal heart failure. In addition, the presence of diffuse parenchymal brain injury or hemorrhage before the procedure, even in the absence of cardiovascular involvement, is a prognostic indicator of a poor outcome. Each case must be analyzed to decide the best approach. In our review, the outcome was negative in newborns who were not eligible for embolization; conversely, more than half of the newborns who underwent embolization survived with good odds of normal development or only mild disability (44–46).

In cases of hemodynamic and brain stability, a pediatric assessment should be performed at least every 2 weeks, with a stable assessment of head circumference, and head CT or MRI should be performed at 4, 8, 16, and 24 weeks (48). Embolization is usually performed around the 5th month (43, 48, 49). This occurs in only 20% of prenatally diagnosed VGMs.

Almost 80% of newborns with VGMs tend to present cardiac insufficiency during the first days of life, with hemodynamic instability being the main cause of death. Lowering pulmonary circle pressure and closing the patent ductus arteriosus (PDA) increase systemic vascular resistance and can trigger cardiac insufficiency. This condition arises due to the volume and pressure overload in the right chambers of the heart. Due to the low vascular resistance in the head, the majority of the ejection fraction is directed toward the brain (the steal effect of VGMs) (40). This phenomenon can cause lactic acidosis and may also cause cardiac and systemic ischemia. Pulmonary hypertension can be present, mainly due to the volume overload in pulmonary arteries (40, 50). On echocardiography, the heart appears structurally normal, but there is cardiomegaly, and the right chambers appear dilated and hypokinetic, while the left chambers are hyperdynamic. Cardiological treatment usually involves diuretics and volume restriction to reduce preload, as well as vasoactive agents, including low-dose epinephrine, dobutamine, and other inotropic agents. In particular, milrinone and levosimendan have been proposed and have shown good results (41, 50–53). A pediatric cardiologist plays a main role in VGM management, and during hospitalization, especially after the embolization, daily assessment is mandatory.

### Outcomes

From our review, the overall mortality of prenatally diagnosed VGMs was almost 60%; however, favorable neurological development was achieved in the majority of survivors. Our results are in line with previous studies (3, 29, 42, 43, 46, 54). It is essential to follow up on the neurodevelopment of these patients by evaluating their milestones using approved tools, such as Bayley Scales of Infant or Toddler Development3rd Edition (BSID-III), and neuro-electrophysiological assessments (54).

### Genetics

In the genetic era, it is recommended that all VGM patients should receive a genetic evaluation.

By performing genome-wide linkage analysis, mutations in RASA1, ENG, ACVRL1, ALK1, SMAD4, and EPHB4 have been associated with genetic VGMs. A specific next generation sequencing (NGS) panel for genetic mutations associated with VGMs should be offered prenatally or after birth. Autosomal dominant mutations of RASA1 gene are responsible for almost one-third of VGM cases, followed by EPHB4, which accounts for 10% of VGM cases (2, 54–57). RASA1 encodes for RAS GTPase activating protein1, which enhances the intrinsic GTPase activity. Studies conducted on RASA1 knockout mice have demonstrated how this gene is fundamental to embryonic vessel formation. Heterozygous mutations leading to the loss of function of EPHB4 are linked to genetic non-immune hydrops fetalis in cutaneous capillary malformation-arteriovenous malformation syndrome and recently to VGM development. This is not surprising since EPHB4 is essential for venous development and differentiation (57). ALK1 is a TGF-β receptor that interacts with endoglin encoded by ENG to activate SMAD4; this is one of the main pathways for angiogenesis. Mutations in ALK1 result in type 2 hereditary hemorrhagic telangiectasia; mutations in ENG and SMAD4 result in vascular malformation (54–57).

### Future prospective

As reported previously, the transition from fetal to postnatal circulation represents a critical point for VGM patients. To date, few case reports dealing with fetal endovascular treatment with interesting results have been published. Naggara et al. presented a case of a 31-week and 4-day fetus with a novel diagnosis of the VGM. At that time, the fetus exhibited cardiomegaly without heart failure, and the MRI performed at 34 weeks and 5 days revealed a mediolateral diameter of the dilated falcine sinus at its narrowest section measuring 10.4 mm. A transutero embolization was performed using detachable platinum coils at 33 weeks without any complications; this procedure resulted in a reduction of the cardiomegaly and the dilatation of the falcine sinus. Labor was induced at 38 weeks. However, on day 5 of life, an urgent transfemoral arterial endovascular approach was needed for acute heart failure; a second procedure was performed at 2 months of life. At 11 months, normal neural development was observed (58). Orbach et al. presented a case of ultrasound-guided percutaneous, transuterine, transcranial embolization performed on a fetus at 34 weeks and 2 days of gestation. The indication for intervention was a dilated falcine sinus associated with a 99% likelihood of neonatal decompensation. The procedure was uneventful, resulting in a 43% reduction in total cardiac output and a reduction in the caliber of the prosencephalic varix and falcine sinus. However, 2 days later, labor started following premature rupture of membranes. The neonatal outcome was good, and after 30 days, no cardiovascular support was needed (59).

## Conclusion

Our study provides data on the prenatal main characteristics of VGMs and includes information regarding prenatal, birth, and postnatal management. To the best of our knowledge, this is the largest review conducted to date. We highlight the importance of personalized management based on neurological and cardiological status. However, our study is limited by the small sample size, primarily due to the rarity of the disease. Also, variability in equipment and medical possibilities across different studies from which the data were extracted presents another limitation. We aimed to offer gynecologists, neonatologists, cardiologists, and neuroradiologists a uniform guide for managing VGMs.

## Data Availability

The raw data supporting the conclusions of this article will be made available by the authors without undue reservation.
